# Research on the ecology of ticks and tick-borne pathogens—methodological principles and caveats

**DOI:** 10.3389/fcimb.2013.00029

**Published:** 2013-08-08

**Authors:** Agustín Estrada-Peña, Jeremy S. Gray, Olaf Kahl, Robert S. Lane, Ard M. Nijhof

**Affiliations:** ^1^Faculty of Veterinary Medicine, Department of Animal Pathology, University of ZaragozaZaragoza, Spain; ^2^UCD School of Biology and Environmental Science, University College DublinDublin, Ireland; ^3^Tick-radar GmbHBerlin, Germany; ^4^Department of Environmental Science, Policy and Management, University of CaliforniaBerkeley, USA; ^5^Institute for Parasitology and Tropical Veterinary Medicine, Freie Universität BerlinBerlin, Germany

**Keywords:** ticks, tick-transmitted pathogens, methodological gaps, protocols, interpretations

## Abstract

Interest in tick-transmitted pathogens has experienced an upsurge in the past few decades. Routine application of tools for the detection of fragments of foreign DNA in ticks, together with a high degree of interest in the quantification of disease risk for humans, has led to a marked increase in the number of reports on the eco-epidemiology of tick-borne diseases. However, procedural errors continue to accumulate in the scientific literature, resulting in misleading information. For example, unreliable identification of ticks and pathogens, erroneous interpretations of short-term field studies, and the hasty acceptance of some tick species as vectors have led to ambiguities regarding the vector role of these arthropods. In this review, we focus on the ecological features driving the life cycle of ticks and the resulting effects on the eco-epidemiology of tick-transmitted pathogens. We review the factors affecting field collections of ticks, and we describe the biologically and ecologically appropriate procedures for describing tick host-seeking activity and its correlation with environmental traits. We detail the climatic variables that have biological importance on ticks and explain how they should be properly measured and analyzed. We also provide evidence to critically reject the use of some environmental traits that are being increasingly reported as the drivers of the behavior of ticks. With the aim of standardization, we propose unambiguous definitions of the status of hosts and ticks regarding their ability to maintain and spread a given pathogen. We also describe laboratory procedures and standards for evaluating the vectorial capacity of a tick or the reservoir role of a host. This approach should provide a coherent framework for the reporting of research findings concerning ticks and tick-borne diseases.

## Introduction

Ticks are fascinating vectors of many pathogens affecting human and animal health, and research on this topic was boosted by the emergence of the zoonotic tick-borne disease, Lyme borreliosis, three decades ago (Ostfeld et al., 2005). The discovery of formerly unknown mechanisms of pathogen transmission, such as the non-viremic transmission of TBE virus (Labuda et al., 1993), and the re-emergence of certain tick-borne diseases, such as the ongoing epidemic of Crimean-Congo hemorrhagic fever in Turkey, likewise have created a wealth of research interest (Gale et al., 2010).

The adoption of DNA detection as a general tool during the 1990s to investigate the relationships between ticks, tick-transmitted pathogens and their vertebrate hosts further accelerated interest in the field. However, this has resulted in much misinterpretation of the vectorial capacity of ticks and the reservoir competence of vertebrates. A review of digital bibliographical databases for the period 2000–2010 revealed 512 papers dealing with ticks and transmitted pathogens in Europe alone (Estrada-Peña et al., 2013). Of these, 311 reported associations and relationships between tick and pathogens, at different scales (local, regional, or national). Further, 109 reports in that period dealt with newly determined associations between pathogens and ticks collected while feeding on a host. As noted by Kahl et al. (2002) “partially or fully-fed ticks removed from hosts that contain [pathogens] may or may not be vectors because nearly all hematophagous arthropods feeding on reservoir hosts are likely to ingest some microorganisms with the blood meal.” Such reporting of crude associations between pathogens and engorged ticks has only served to confuse our understanding of the relationships between ticks, tick hosts and tick-transmitted pathogens.

A later surge in interest in ticks and tick-borne pathogens has been inspired by recent claims about the impact of forecasted climate change on the spatial distribution of ticks and associated pathogens (Brownstein et al., 2003; Ostfeld et al., 2005; Diuk-Wasser et al., 2006; Ogden et al., 2008; Jaenson et al., 2009). However, this research has been fraught with difficulty from the outset because of insufficient knowledge about the nature of many tick-pathogen associations (Randolph, 2009; Franke et al., 2013; Medlock et al., 2013). A proper understanding of how abiotic factors shape the transmission cycles of tick-transmitted disease agents awaits a more rigorous analysis that is often limited by the current availability of data and the many indirect mechanisms that bear on them (Kahl et al., 2002; Eisen, 2008).

We assume that the many procedural and analytical errors in current tick and tick-borne zoonotic disease research are often a consequence of a lack of knowledge or of suitable training. This paper is not intended as an exhaustive review on tick biology and behavioral ecology, but a summary of existing knowledge regarding the processes involved in planning surveys, the collection of field data, and the relationships of ticks with abiotic variables. We have paid special attention to the variables regulating the activity of the ticks, and how these should be recorded and interpreted. Further, we discuss the need to standardize the eco-epidemiological terminology of ticks, hosts, and pathogens, as already discussed by Kahl et al. (2002) among others. We focus on exophilic ixodid ticks, which are those that develop on the ground, without needing to shelter in burrows or nests of their vertebrate hosts (such ticks are referred to as endophilic).

## Overview

**Figure 1:**
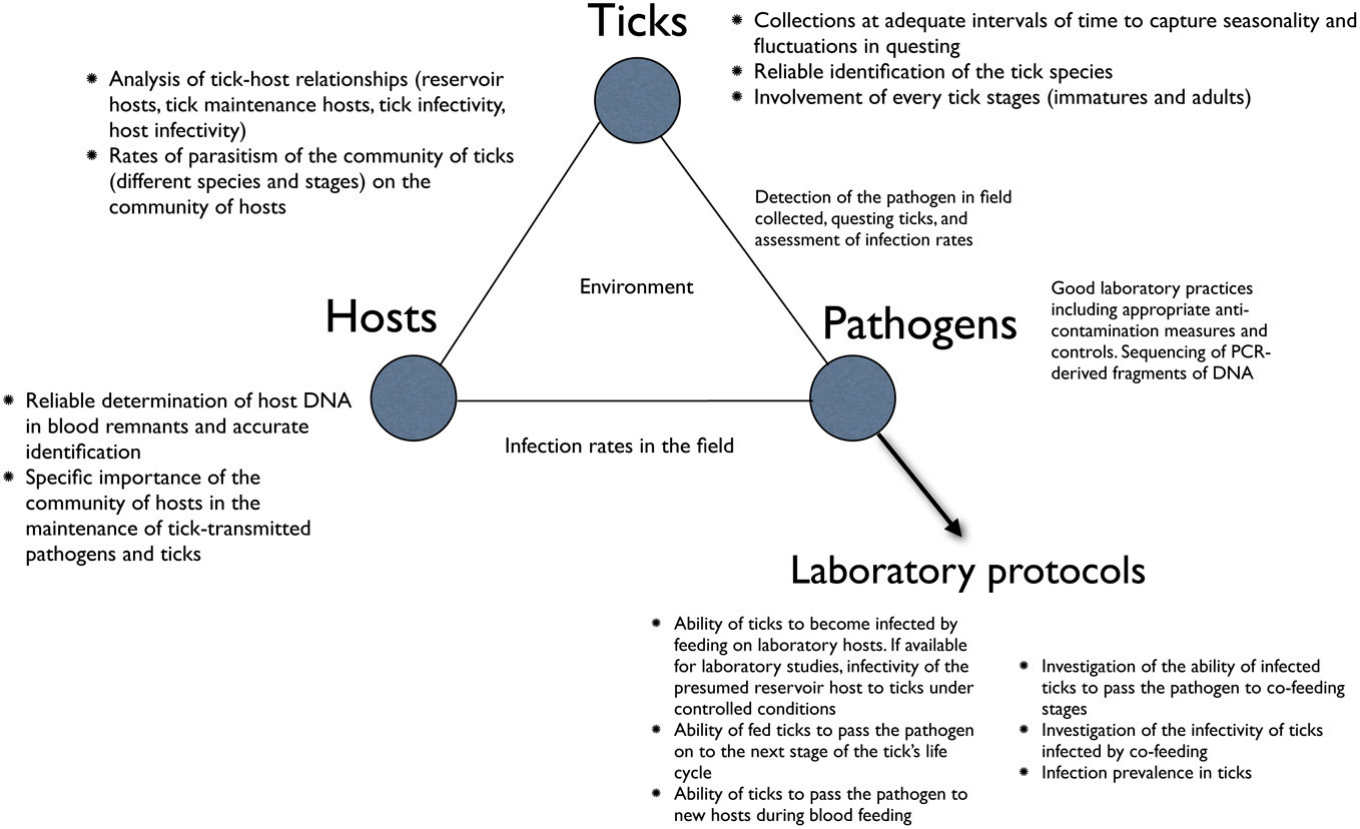
**A general description of the main points to address when studying the relationships between ticks, host and pathogens.** The chart is aimed to pinpoint the key steps in these relationships and to outline the most common procedural errors detected in the scientific literature.

Figure 1 summarizes the flow of information and critical points in the collection of ticks, and the determinations and interpretations of relationships between ticks and microorganisms, which we will address in this review. The three key elements in this ecological system, namely the ticks, the hosts, and the tick-transmitted pathogens are impacted directly or indirectly by abiotic and biotic factors. We will review what specific combinations of abiotic factors activate ticks, and how to collect questing ticks to minimize sampling bias and maximize the robustness of the resultant data. We further review how to correlate weather variables with empirical data on ticks. At each stage of the surveys, accurate identification of ticks, their vertebrate hosts and associated microorganisms is critical, because errors would lead to reports of unusual associations of vertebrates, pathogens and ticks. Every tick specimen should be identified with reliable keys and compared with voucher reference specimens when necessary, before extracting their DNA for molecular analyses. In the same way, the lack of reliable identification of the hosts, or the erroneous detection of host DNA in the blood-meal remnants in the tick, might prevent accurate determination of the associations between the three partners. In the case of pathogen detection, polymerase chain reaction (PCR) and other molecular assays must be done in strict accordance with good laboratory practices (GLPs) and must include positive and negative controls with each run.

The importance of laboratory work is also shown in Figure 1. While the prevalence of a given pathogen may be calculated from field collections of unfed ticks, the vector status of the tick cannot be determined from such data alone. Additional vector and reservoir competence studies must be carried out in the laboratory before it can be concluded that a particular tick is a vector, or a host is a reservoir of a pathogen. We elaborate on these concepts in a later section of this review.

## The collection of questing ticks

A critical initial step in studies on ticks and associated pathogens is the collection of the ticks, which, for the production of reliable results, should adhere to a series of principles. The abundance of ticks can be estimated either by surveying the hosts on which ticks feed or by sampling low vegetation or leaf-litter areas at defined intervals and periods of time. A rough estimation of the density of questing ticks can be obtained by sampling questing ticks from the vegetation and from the leaf litter (Tälleklint-Eisen and Lane, 2000b). Additionally, this approach can also be used to procure ticks for testing for zoonotic agents. Questing is the process in the life cycle of ticks in which they actively seek a host. The span and the intensity of the periods of questing, and even the simultaneous questing of different stages of the same species, may have profound epidemiological implications for the epidemiology of particular tick-transmitted pathogens (Randolph, 2000). By adequately recording the changing abundance of questing ticks, the response of a species to weather traits can be estimated, and their geographical or seasonal changes, tracked.

The common procedure for collections of questing ticks is by dragging a piece of fabric such as flannelette or blanket across the vegetation surface or over the leaf litter. These can either be large pieces of fabric dragged behind the operator, or more commonly are relatively small pieces (1 m^2^) mounted on poles and brushed over the vegetation, a procedure known as “flagging.” Many operators compromise between these two approaches. The ticks adhering to the fabric are collected at varying intervals, but it should be borne in mind that prolonged dragging distances result in a loss of ticks (see below). Flagging (or dragging) can provide an estimate of the rates of tick activity, which are characterized by several cycles of ascending and descending movements of the ticks in the vegetation. It is necessary to follow some basic rules in order to obtain an accurate representation of the questing activity, so that comparisons may be made with data from different sites, periods of time and from other studies, and to associate the observed questing rates with abiotic features (e.g., weather). Sampling must be conducted over homogeneous patches of vegetation, but a stratified sampling approach may be adopted to obtain a reliable representation of questing ticks in a given area. Vegetation cover affects the efficiency of the flagging or dragging method and heterogeneously modifies the micro-climate at the level where the ticks quest, thereby affecting their behavior and their observed abundance (Tälleklint-Eisen and Lane, 2000b). It also affects the abundance of hosts, and this may influence the perceived abundance of ticks, at the scale of small patches of vegetation. Tick abundance may vary significantly by aspect (exposure, such as south-facing) in hilly or mountainous areas (Lane et al., 1985).

To determine tick density (abundance per unit area) it is necessary to walk slowly over a defined distance. In some sites it is impossible to obtain sufficient uniformity for area-based dragging, and a time-based approach is indicated (e.g., number per hour) with adequate standardization. There are no rules regarding the overall length of a transect, but small ones (<100 m) may adversely affect the reliability of the results, since the spatial distribution of ticks is usually highly aggregated, even at sites where weather conditions and vegetation appear to be homogeneous (Wilson, 1998; Tälleklint-Eisen and Lane, 1999; Randolph, 2000). For each transect the operator must stop frequently enough (e.g., every 10 m) to remove ticks from the flag or drag otherwise too many ticks will be dislodged by the substrate. Sites with a high density of ticks demand more frequent removal of ticks from the fabric. The results should preferably be expressed as the number of ticks per unit area, but occasionally sites are too difficult to sample in this manner and in these cases a standardized time-based approach may be used (Gray et al., 1992). It also should be borne in mind that only a relatively small proportion of the total population of ticks in a given area may be questing simultaneously.

The questing activity of some ticks cannot be determined by dragging. For example, the immatures of some species of *Dermacentor,Rhipicephalus*, and *Hyalomma* are mainly endophilic, living inside the burrows of their hosts (Salman and Estrada-Peña, 2013). This also applies to certain species in the genus *Ixodes* that are nidicolous (Filippova, 1977; Lane et al., 1999). The only way to evaluate the seasonality of endophilic ticks is by direct sampling of the nesting materials or the burrows of their hosts or the primary hosts themselves (Vial, 2009). Moreover, the adults of the genera *Amblyomma* and *Hyalomma* are not collected homogeneously by dragging, because they have a “hunter” strategy to find a host. These ticks tend to shelter in litter areas to escape from desiccating microclimate conditions, and although they may be collected by dragging, this method is inefficient for such species. A survey should involve at least two or three consecutive years of sampling at relatively short intervals of time to be meaningful, preferable at 7–10 day intervals. Budgetary or other constraints may render this difficult, but relatively infrequent sampling (e.g., monthly intervals) fail to capture the seasonality of most tick species as well as the influence of abiotic factors on questing.

Quantitative terms regarding questing ticks have been reviewed previously (Wilson, 1994; Kahl et al., 2002). The *abundance* of questing ticks is a dimensionless measure, whereas *density* is a description of the number of ticks collected per unit area or time (Kahl et al., 2002). It is a mistake to interpret the number of questing ticks collected in field surveys as a measure of the total number of ticks in an area (Tälleklint-Eisen and Lane, 2000a,b).

Another method to obtain estimates of relative tick abundance is sampling from hosts. Ideally sentinel domestic animals such as sheep should be used in defined areas, but this is not often possible and most data sets consist of samples from domestic animals in relatively uncontrolled circumstances, from trapped rodents or birds and from wild ungulates at hunting points. Complete data sets are usually not possible because of the preferences of different tick stages for certain host species. Another drawback of this approach may be the fluctuations of host abundance, usually outside the control of the investigator that affect the intensity of tick infestations. However, compared with the sampling of ticks from the vegetation and leaf litter, data from animals are not so affected by short-term variables such as weather or from factors that can distort the data such as the structure of vegetation. When ticks are collected from hosts, the *prevalence of infestation* describes the percentage of hosts examined and found infested. The *mean density* of ticks per host is calculated as the total number of ticks obtained, divided by the total number of hosts examined. The *intensity of infestation* is the total number of ticks observed divided by the number of infested hosts (Kahl et al., 2002).

## Relating the phenology of ticks to environmental (abiotic) features

Tick researchers have been interested in discovering what abiotic factors drive the biology of ticks. The potential exists of evaluating seasonal patterns of activity resulting from various combinations of climatic conditions and in thereby obtaining an estimation of “risk” in a climate change scenario (Mannelli et al., 2003). Many reports have correlated environmental traits with empirical data concerning the life cycle of several species of ticks. Results obtained so far confirm that the development and questing of *I. ricinus* (Lees and Milne, 1951; Gray, 1982, 1991; Perret et al., 2000, 2003, 2004; Randolph, 2002; Randolph et al., 2002; Estrada-Peña et al., 2004), *I. scapularis* (Daniels et al., 1996; Lindsay et al., 1999; Ogden et al., 2008), *I. pacificus* (Padgett and Lane, 2001), *A. americanum* (Schulze et al., 2001), *R. appendiculatus* (Randolph, 1993, 1997), *Hyalomma* spp. and *Rhipicephalus* spp. (Petney et al., 1987; Pegram and Banda, 1990), among others, are regulated by weather. It is, however, necessary to understand how the ticks become active before constructing guidelines for evaluating their patterns of activity.

Most ixodid ticks are inactive in the lowest layers of vegetation or in the leaf litter or soil before they begin to quest. A notable exception are the subadults of *I. scapularis*, which nocturnally can locate and attach to lizards sleeping in soil (Lane et al., 1995). The combination of a set of suitable conditions, which normally involves an activation temperature in the spring, triggers the activity causing the ticks to climb to the top of the vegetation to quest for hosts. During questing, ticks may lose water (Lees, 1946) that they normally regain by descending at intervals into the litter zone (Lees and Milne, 1951) where the ticks actively reabsorb water vapor from the atmosphere (Rudolph and Knülle, 1974; Kahl and Alidousti, 1997). After the ticks are rehydrated, they are ready to ascend the vegetation. Ticks vary in their ability to retain or to gain water (Kahl and Knülle, 1988) and there is an interspecific variability in the management of their water balance. Extrinsically, tick water balance is affected by the saturation deficit of water in the air (affecting water loss) and by relative humidity (affecting the possibility of water gain by active water vapor uptake), and, intrinsically, among other points, by the capability of ticks to find places with a favorable microclimate even when the weather is warm and dry, e.g., in the leaf litter. The energy reserves of the tick plus its ability to maintain an acceptable level of body water are the factors mainly regulating the short-term questing behavior of ticks. Host stimuli may also affect tick activity.

Tick questing activity in temperate regions follows a seasonal pattern with some short-term fluctuation largely depending on weather. Some species of ticks may have an almost uninterrupted process of parasitic and questing periods in tropical regions, because of the stable weather conditions (Belozerov and Kvitko, 1965). Besides weather or microclimate, photoperiod regulates tick seasonal periodicity by prevention (*behavioral diapause* in unfed ticks) or stimulation of questing activity in unfed specimens, or by interrupting development (*morphogenetic* or *developmental diapause* in engorged ticks). In both cases the inducing stimulus is the photoperiod, the day-night relative duration (or its change), which is perceived by ticks (Belozerov, 1982). These diapause mechanisms optimize the fate of a given population by enabling the ticks to find a host, to feed and to enter the molting period before the onset of adverse weather conditions such as winter. Although many studies and reviews have discussed how diapause determines the phenology of ticks (Gardiner and Gray, 1986; Pegram et al., 1988; Gray, 1991; Norval et al., 1991; Madder and Berkvens, 1997; Randolph, 1997), photoperiod is often ignored in studies on the seasonality of ticks.

Temperature is one of the main regulators of tick phenology. Only the temperature recorded between the litter and the height where ticks quest should be associated with the empirical data from field plots or dragging surveys. Other series of data, such as those recorded at stations used for climate recording, commonly placed at 2 m above the ground, are not suitable because they do not reflect the actual climate to which the ticks in the field site are exposed. There tends to be smaller variation of temperature and humidity at 2 m above the ground level than in the vegetation layer. The subtle variations of the microclimate experienced by the ticks will almost surely be lost in data series obtained from meteorological stations and possible correlations between weather and tick activity patterns are likely to be obscured. Ideally portable micro-loggers should be placed on the ground litter to record the weather at the sites where ticks develop and quest. A series of micro-loggers can also be placed at different heights above ground level to track changing conditions in microenvironments where the different tick stages quest.

Since parts of the seasonal tick activity pattern can be explained by responses to water loss, variables related to air humidity would probably show a regulatory action on the recorded phenology of questing ticks. It was demonstrated that the questing duration of nymphal *I. ricinus* is inversely related to the saturation deficit, whereas the duration of quiescence is not (Perret et al., 2003). The air water saturation deficit is the “drying power” of the air and is correlated with the losses of water by living tissues (Anderson, 1936). When environmental conditions are less desiccating, ticks will quest for longer periods. Abrupt declines in the proportion of questing ticks have been shown to coincide with abrupt increases in saturation deficit at field sites in Switzerland (Perret et al., 2000), the United Kingdom (Randolph et al., 2002) and Spain (Estrada-Peña et al., 2004). It is quite possible that other tick species exhibit similar behavior. It is important to note that relative humidity has a critical effect on the ability of ticks to absorb water. There is a critical equilibrium humidity above which ticks can actively absorb water and maintain their water balance (Knülle and Wharton, 1964) and survive for long time periods irrespective of the saturation deficit. Below this threshold of relative humidity, ticks cannot actively absorb water. Therefore, under conditions of low relative humidity, ticks cannot rehydrate even if the saturation deficit is low. However, under conditions of high relative humidity (e.g., >85% in *I. ricinus*) ticks can actively take up water from air and saturation deficit may have a lesser role in the regulation of questing. This is a complex process because temperature, saturation deficit and relative humidity change on a diurnal basis.

Saturation deficit and relative humidity are not the same as rainfall, which is the trait commonly used to explain the course of tick questing activity or the mortality of questing or developing ticks. Patterns of precipitation undoubtedly have an effect on the relative humidity at the regional scale (Rao et al., 1996; Thornton et al., 2000). However, the effects are not the same in different biomes. There is no universal relationship between air humidity (or saturation deficit) and rainfall patterns. It is likely that analysis of rainfall as the only effective mechanism for the mortality or activity of ticks will produce poor correlations and erroneous conclusions.

## Confounding factors and procedural gaps in studies of tick phenology

Multiple factors may affect the phenology of ticks, even if collections are carried out under a harmonized protocol as outlined above. Studies on the seasonality of ticks often correlate questing rates with the weather factors. However, an increase in questing rates may result from independent factors such as recruitment of newly molted ticks or resumption of activity during favorable weather conditions. A variable number of individuals may delay their onset of questing as a result of behavioral diapause (Gray, 1991), and older individuals may survive from previous periods of questing and overlap with later molted specimens. This could confound the correlation of the observed levels of activity with environmental factors. There is a lack of adequate studies regarding the effects of photoperiod on the induction of diapause and thus on tick seasonality (Gray, 2008). In fact, most recent research on seasonal activity has addressed only the presumed actions of either temperature or relative humidity.

One of the common ways to summarize tick questing activity is the clustering of observations by habitat type or plant alliances. The classification of the alliances of plant species into ecological categories is an old discipline that provides a homogeneous classification of the world biomes, which can then be correlated with weather traits and the characteristics of the soil (Menzel et al., 2006). Categories of vegetation are built on rules that summarize the dynamics of the plants and their response to the prevailing features of climate. These associations are in any case coarse in scale and the pattern emerges at national or continental scales. The association of the distribution patterns of ticks throughout the dominant biomes in large regions has been widely used to deal with otherwise confusing information about the distribution of ticks (Gilot et al., 1992, 1996). However, such correlations might give the false impression that the finely-tuned local processes of tick phenology are consistently correlated with simple categories of vegetation. Analyses of life cycle processes require linking the scales at which variation is measured to the scales at which the processes operate (Huston, 1999): The dominant vegetation influences occurrence, development and activity of ticks because it modulates the microclimate and because it affects the abundance of hosts. The actual driver of the processes is the microclimate, and the vegetation is only an indicator (Randolph and Storey, 1999). We advocate the description of patterns of presence and abundance of ticks as related to the vegetation only after understanding how *local* vegetation affects *local* microclimate and thus its indirect effects on the processes of tick life cycles. Otherwise, considerable information is lost in the process of clustering into categories of vegetation.

## Identification of ticks

The importance of the correct identification of ticks is commonly underestimated. Without accurate identifications, every conclusion drawn from such material would be erroneous. Although ticks have attracted much interest in the past few years, this interest has not necessarily been accompanied by an adequate taxonomic expertise. The taxonomy of ticks is a rather complex topic, which is still the subject of debate. The identification of ticks is still mainly (if not only) based on the morphology of the specimens and on individual expertise, with the support of a variety of keys that describe the morphology of the stages and the differences between closely related species. Publications that cover every species in a particular area or country are seldom available, with a few notable exceptions (Filippova, 1977, 1997; Keirans and Clifford, 1978; Matthysse and Colbo, 1987; Durden and Keirans, 1996; Walker et al., 2000). A literature search on the epidemiology of tick-transmitted pathogens published in the past few decades suggested that few of the authors had taxonomic expertise, and there is usually no indication of how the ticks in their studies were identified. Contemporary researchers tend to use convenient, though not always relevant, reprints (available for example in their institute library or on the internet). This approach reveals a lack of criticism in a critical step of their research. It has become customary to identify only the adult stage of ticks, probably because it is easily collected from domestic hosts and easier to identify that the immatures. However, this provides an unreliable overview of the diversity of ticks present and their relative importance in the community of hosts and pathogens.

Adequate identification methods based on DNA or proteome analysis of ticks such as barcoding and mass spectrometry, are under development (Lv et al., 2013; Yssouf et al., 2013), but much work is still necessary to verify their significance. The complete genome has been sequenced for only *I. scapularis* (Pagel Van Zee et al., 2007), and the reliability of the sequences of other species deposited in GenBank is uncertain, as has proved to be the case for other taxa (Bridge et al., 2003; Wägele et al., 2011). Knowledge about the intra- and interspecific variation of the few molecular sequences so far examined is limited and therefore the significance of sequence variation is difficult to interpret. At present, identification of ticks based only on molecular data should be discouraged, and while a potential source for future research, sequences available in GenBank should not be considered as the “gold standard” for tick identification.

## Identification of tick hosts by determining the source of tick blood-meals

Another biotic component of these systems are the hosts. Several studies have addressed the importance of examining the complete community of hosts in a territory to estimate their relative contribution in supporting the various tick species present and the pathogens the ticks transmit (Levin and Fish, 1998; Ostfeld and Keesing, 2000; Logiudice et al., 2003; Tyre et al., 2003). In sylvatic habitats the accessibility of the hosts is an obvious problem and determination of their role in the maintenance of ticks and pathogens has traditionally required expensive and exhaustive trapping or shooting. However, several variations on a method to detect host DNA in the remnants of blood within questing ticks are now available. These consist of amplifying fragments of conserved genes such as *cytochrome b* (Kirstein and Gray, 1996); 18SrRNA (Pichon et al., 2003) and 12SrRNA (Humair et al., 2007), followed by reverse line blot (RLB) or sequence analysis. Some success has been achieved in determining the role different host species play in maintaining populations of *Ixodes ricinus* and in identifying possible reservoir hosts of *Borrelia burgdorferi* sensu lato (Estrada-Peña et al., 2005; Pichon et al., 2005; Moran Cadenas et al., 2007), but the method has proved highly susceptible to contamination and sensitivity problems. While there is impetus for such an approach, at present it cannot be considered sufficiently robust for general deployment in field studies. Recently developed methods to identify the blood meal source in questing ticks using proteome profiling techniques (Wickramasekara et al., 2008; Onder et al., 2013) or natural isotope analysis (Schmidt et al., 2011) may be interesting alternatives.

## Evaluation and standard denominations of ticks-hosts-pathogens relationships

The interactions between the elements of any tick-host-pathogen system and interactions with other transmission cycles are key features to understand pathogen prevalence in questing ticks and in vertebrates. The evaluation of such interactions is a necessary preliminary (though complex) step in risk assessment. There are several reviews addressing the survival strategies of tick-transmitted pathogens, and how the ecology of ticks and their hosts may shape such prevalence (Mannelli et al., 2012), but these aspects are beyond the scope of this review. It is necessary to summarize a framework of relevant ecological terminology proposed by Kahl et al. (2002) that was largely based on terms introduced by Pavlovsky (1966), and continued by Balashov (1972), Ginsberg (1993); Nuttall and Labuda (1994); Sonenshine and Mather (1994), and Randolph and Craine (1995). Such a framework has been constructed to address major pitfalls in ecological research, identifying common errors and faulty interpretations occurring in the literature.

The eco-epidemiological status of ticks and their hosts cannot be determined by field studies alone. Complementary laboratory studies should be designed to capture the essentials of the role played by each element of the system. Some hypotheses can only be confirmed after tests have been conducted in the laboratory to remove the inherent variability of the field data. To designate a tick as a vector of a given pathogen, the three following criteria must be confirmed:

1. Ability of ticks to become infected by feeding on the infected hosts. Whenever possible these studies should be carried out using uninfected ticks and natural, presumed reservoir hosts.
2. The ability of fed ticks to trans-stadially pass the pathogen through the following molt to the next stage of the life cycle, or the F1 generation.
3. The ability of molted stages to transmit the pathogen to naive hosts during blood feeding.

In summary, to qualify as a vector, the tick must (1) feed on infectious vertebrates (2) acquire the pathogen during the blood meal (3) maintain the pathogen through one or more trans-stadial molts and (4) transmit the pathogen to previously unexposed hosts while feeding again (Kahl et al., 2002). Assigning vector or reservoir status to a tick or a host species based solely on the detection of the DNA of the pathogen or evaluation of hosts by serology is not acceptable. Detection of pathogen DNA in a tick or host indicates that it possesses *carrier* status (see below for the definitions), and detection of antibodies in host sera merely indicates that an animal has been exposed to the pathogen. Whether or not transmission from tick to host or from host to tick is involved must be addressed experimentally in the laboratory.

**Figure 2:**
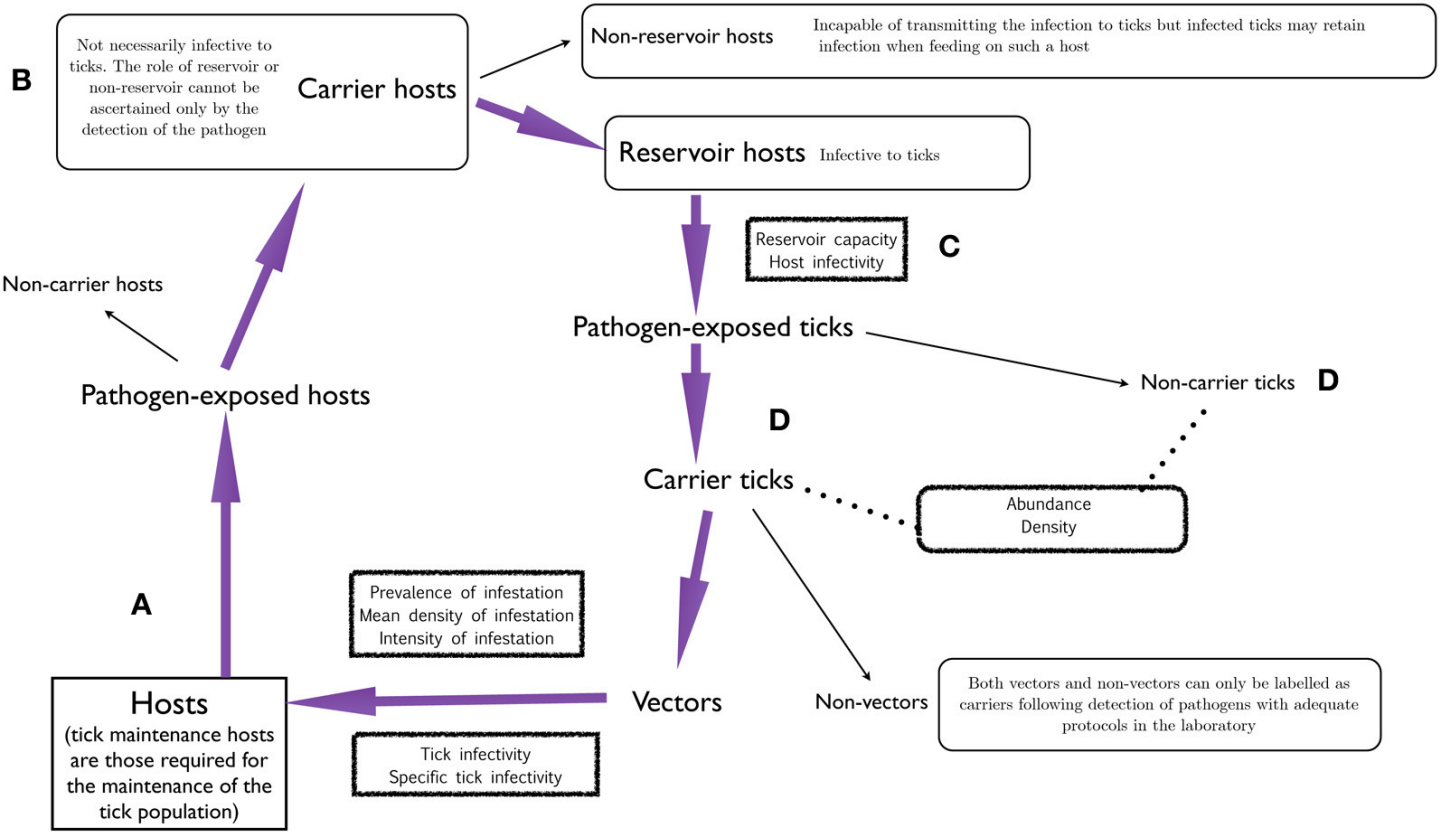
**A chart describing the chain of events in the interface of ticks, hosts, and pathogens, aimed to delineate a set of standard denominations following a common framework (Kahl et al., 2002).** Hosts **(A)** may be exposed to pathogens and then be carrier hosts **(B)**. These may be reservoir hosts **(C)** that feed ticks exposed to pathogens. The ticks feeding on these reservoir hosts may be the carrier ticks **(D)** that are vectors of pathogens to new hosts.

Figure 2 outlines the steps in the chain of host-tick-pathogen relationships. Tick hosts (Figure 2A) include all vertebrates on which ticks feed in nature. Local and regional tick abundance largely depends on the abundance of *tick maintenance hosts* (sometimes called *amplifying hosts*, a term also applied occasionally to vertebrates that transmit pathogens and in which the pathogens replicate). The lack of sufficient numbers of hosts or even the absence of a key host in a particular area may prevent the occurrence of a species of tick even if weather and other abiotic features are permissive. When hosts are fed on by *infected vector ticks* they may be *pathogen-exposed* and these hosts may be *carriers* or *non-carriers*. The latter cannot infect naive ticks. A carrier host (Figure 2B) is not necessarily infective to ticks and therefore the terms *non-reservoir* and *reservoir* must be used to define infectivity status. The former are incapable of transmitting the infection to uninfected ticks. There are two important terms that apply to reservoir hosts and are relevant as to how ticks feeding on them are exposed to pathogens (Figure 2C). *Reservoir capacity* describes the absolute contribution made by a particular reservoir host species to the natural prevalence of infection by a given pathogen within a certain site. *Host infectivity* denotes the efficiency with which the host transmits the infection to ticks feeding on it. Reservoir capacity is dependent upon its infectivity for feeding ticks and the duration of the infective period (Mather et al., 1989; Mannelli et al., 1993; Randolph and Craine, 1995; Kahl et al., 2002).

By feeding on reservoir hosts, ticks are exposed to pathogens and they may become *non-carrier* or *carrier* ticks (Figure 2D). Carrier ticks may be *vectors* or *non-vectors*, as already discussed. The terms *tick infectivity* and *specific tick infectivity* refer to the efficiency with which the infection is transmitted from ticks to hosts and the efficiency with which the infection is transmitted from a given tick species to a particular species of host, respectively, and these features must be addressed in the laboratory.

## Molecular diagnostics of tick-borne pathogens

For all eco-epidemiological studies on tick-borne diseases, correct identification of the tick-borne pathogen(s) in question is pivotal. Examples of conventional methods for the identification of pathogens include cell culture, immunofluorescence and microscopic techniques (Kahl et al., 2002), but the detection of pathogens was revolutionized following the advent of the polymerase chain reaction (PCR) in the mid-1980s. The PCR made it possible to amplify minute amounts of (pathogen) DNA from biological samples such as ticks or blood for subsequent analysis by means of gel electrophoresis, restriction fragment length polymorphism (RFLP), sequencing, or hybridization probing techniques such as the RLB (Persing et al., 1990; Rijpkema et al., 1995; Richards, 2012). Quantification of the copy number of pathogen DNA initially present in tick or host samples became reality following the development of real-time PCR, in which the amount of product formed during the PCR reaction can be monitored (Pusterla et al., 1999).

In general, a well-designed PCR assay has a superior sensitivity and specificity vs. direct-detection methods, such as visual inspection of blood or tick hemolymph smears. It also offers the advantage over serological methods in that it can document current infection in ticks and animals and not simply past exposure to a pathogen, and does not require adaptation to different animal species, which is particularly useful in the examination of samples collected from wildlife. Unfortunately, the widespread use of PCR has led to a tendency to incriminate tick species as vectors based solely on the detection of pathogen DNA. Although the screening of ticks for pathogen DNA by PCR may be useful to identify potential tick vectors or reservoir vectors and to determine the tick-borne pathogens which circulate in a defined area, the findings must always be interpreted with great caution, as positive results may be caused by remnants of imbibed blood meals containing pathogen DNA, which does not necessarily implicate the tick as a vector (Kahl et al., 2002). This is further illustrated by experimental studies in which pathogen DNA was detected by PCR in non-vector species that had fed on infected animals as larvae, but were not capable of transmitting the pathogen *in vivo* (Des Vignes et al., 1999; Soares et al., 2006). To circumvent this problem, an alternative approach is to detect pathogens or pathogen DNA in the salivary glands of ticks. It should, however, be noted that certain pathogens such as *B. burgdorferi* s.l. are restricted primarily to the midgut of infected ticks and migrate to the salivary glands only during feeding (De Silva and Fikrig, 1995); pathogen DNA of *B. burgdorferi* s.l. is therefore unlikely to be found in the salivary glands of unfed ticks. For the tick-borne bacterium *Anaplasma centrale*, it has been demonstrated that colonization of the salivary glands does not prove that a tick can transmit the pathogen to a host; the salivary glands may act as a distinct barrier for the efficient pathogen transmission (Ueti et al., 2007). Hence it is necessary to perform transmission experiments to confirm the vector capacity of a given tick species.

Following collection and the correct identification of ticks, the first step in the laboratory is the extraction of nucleic acids. Collected ticks can be used directly for DNA extraction, stored in 70% ethanol or cryopreserved (Mtambo et al., 2006). Since PCR inhibitors can be present in engorged as well as in unfed ticks (Schwartz et al., 1997), DNA extraction methods which are proven to eliminate inhibitors from the purified DNA template should be used. Other methods to overcome PCR inhibition are reviewed elsewhere (Rådström et al., 2004). Ticks should be clean and free of debris such as plugs of cement prior to homogenization or dissection. Live or freshly killed ticks can be dissected to isolate tissues such as salivary glands for subsequent DNA extraction. Pictorial guides on commonly used methods to collect tick tissues, saliva or hemolymph were published recently (Edwards et al., 2009; Patton et al., 2012). When DNA is extracted from tick tissues, adequate cleaning of materials between processing individual samples and washing of tissues is required to rule out carry-over contamination between different ticks and tissues.

When designing PCR experiments, it is important to prevent the unspecific amplification of DNA. Since the nucleotide sequences of primers presented in scientific papers are not always accurate or may be outdated when novel nucleotide sequence information for related species has become available (Baker et al., 2003), it is advisable to check the specificity of the primers which are to be used. This can for instance be done using BLAST (http://blast.ncbi.nlm.nih.gov/). Similarly, the design of oligonucleotide probes used for hybridization assays such as the RLB also requires critical appraisal and validation, to ensure their specificity and prevent cross-reactions that may lead to false-positive results (Nagore et al., 2004; Bhoora et al., 2009).

The high sensitivity of PCR and other PCR-based technologies such as loop-mediated isothermal amplification (LAMP) renders it prone to false-positive results due to exogenous contamination. Guidelines to avoid contamination by following the principles of GLP have been published and also include a strict spatial separation of pre-PCR and post-PCR activities, a unidirectional workflow, decontamination measures, and sufficient and adequate negative controls (Lo and Chan, 2006). An additional measure that can be taken to prevent carry-over contamination of PCR amplicons formed during previous PCRs is the use of dUTPs with Uracil DNA Glycosylase (UDG) which renders uracil-containing DNA from previous PCR reactions incapable of being copied by Taq DNA polymerases (Longo et al., 1990). The dUTP/UDG protocol is not compatible with proof-reading DNA polymerases. Read-out of results from certain LAMP tests can be done without opening the reaction tubes, which also reduces the chances of amplicon contamination (Francois et al., 2011).

Whatever method is used for post-PCR analysis, it remains important to use common sense when analyzing the data. Unconventional results in particular have to be verified, and visual inspection of blood smears is often essential, even in the post-genomic era.

## Mathematical models applied to capture the niches of ticks from surveys

Climate change and climatic variations within seasons are likely to influence the epidemiology of vector-borne diseases (Patz et al., 2003). There are reports on field observations of the spread of ticks in several areas of the world (Tälleklint and Jaenson, 1998; Daniel et al., 2003; Materna et al., 2005; Ogden et al., 2008; Jaenson and Lindgren, 2011; Madder et al., 2012). This has kindled interest in capturing the basic patterns of climate and other environmental features regulating the geographical ranges of ticks and their associated pathogens. Since this is a growing discipline in the field, we only outline some misconceptions that may affect the performance and conclusions drawn from modeling exercises.

The capture of data to estimate the direct and indirect effects of weather on the distribution, phenology or spread of ticks is commonly based on the idea of “climate” or “environmental” niche (Soberón and Nakamura, 2009). This is defined as the “intersection” of values of the climatic variables at which optimal development and mortality occur, resulting in the best performance of the population. This concept assumes that the most important factors driving the performance of ticks are related to weather and that niches can be reconstructed by relating data on the occurrence of the tick with datasets summarizing climate, topographic, edaphic, and other “abiotic” or “ecological” variables. It is also assumed that (1) there is a *niche conservatism* and the organism tracks the sites where adequate conditions exist (not taking into account adaptations of local populations to local weather) and (2) the complete distribution range of the organism has been surveyed. The tick's niche is evaluated to *infer* its associations with environmental variables. Inference is later *projected* into a target area to obtain a map that explains “how similar” the conditions in space are, compared with the ones where the tick has been collected. Such a measure of similarity is not an estimate of abundance or of distribution, although it is incorrectly assumed to be a “risk map,” in which “climate similarity” is interpreted as a direct estimator of abundance. Many other variables, including the fine scale distribution of hosts, human factors altering the habitat, geographical barriers to host movements and the uncertain effects of the vegetation, influence the abundance of ticks (Estrada-Peña et al., 2012) and therefore the crude map is not a direct projection of the spatial risk.

Other procedural gaps in the evaluation of the environmental niche of a tick involve the inadequate utilization of interpolated climate datasets, which inflate the models because of autocorrelation (Legendre, 1993) and colinearity (Storch et al., 2003). Both factors strongly modify the apparent influence of variables that affect the distribution of an organism, resulting in the false perception of a well-fitted model. Determination of climate niche is also affected by the partial and subjective use of a limited number of collections of the tick to be modeled (see Porretta et al., 2013), by the inclusion of variables that are correlated with others to build the model, such as altitude and temperature (i.e., Diuk-Wasser et al., 2006), or by the selection of variables based on the predictive performance of the best model, but lacking a biological significance [as mentioned by Braunisch et al. (2013)]. The utilization of physical altitude or elevation in attempts to relate tick occurrence patterns to environmental variables is misconceived. It is obvious that, at a local scale, elevation greatly affects the patterns of climate, decreasing the temperature over the altitudinal gradients and influencing the rainfall. In every case the performance of the model will be inflated giving the false perception of a robust model, biasing the conclusions and the projections. Because of the structure of the algorithms explaining the distribution of an organism (Elith et al., 2010), the use of covariates such as rainfall will produce a false estimate of a good fit, apparently covering the known distribution of the tick. It will, however, be a fortuitous correlation, and the response of the distribution of the tick will not parallel changes in rainfall.

## Conclusions

Increasing interest in tick-borne zoonotic agents, accompanied by the development of accurate and accessible molecular tools, has resulted in a marked upsurge of published papers on the topic over the past three decades. Although this has resulted in distinct progress in knowledge, some of the hard-learned lessons of the past have inevitably been overlooked during this period of intense activity. Typical problems include errors in tick identification (especially of immature instars), premature or erroneous reporting of ticks as new vectors and hosts as reservoirs, superficial data on the abundance and seasonal activity of ticks, inappropriate use of statistical methodology, and uncontrolled laboratory diagnostic procedures. The very ready accessibility of research publications online, at least in abstract form, tends to exacerbate the situation in that, as a result of superficial reading and subsequent citing, an erroneous conclusion can quickly become embedded in the literature. In this review we have described what consensus opinion views as the correct methodologies for obtaining and interpreting data in research on ticks and tick-transmitted pathogens and have highlighted and illustrated some of the common procedural missteps, with suggested solutions where possible. It is hoped that this review will contribute to a more considered and thoughtful approach to this complex topic in the future.

### Conflict of interest statement

The authors declare that the research was conducted in the absence of any commercial or financial relationships that could be construed as a potential conflict of interest.
